# Management of traumatic superficial palmar arch pseudoaneurysm: a therapeutic challenge

**DOI:** 10.1590/1677-5449.202300732

**Published:** 2023-08-14

**Authors:** Patrick Bastos Metzger, Fernando Antonio Falcão Paixão, Sarah Fernandez Coutinho de Carvalho, Miguel Godeiro Fernandez, Simone Lessa Metzger, Maria Fernanda Lima Brandão, Rafael Borges Monteiro, Fabio Henrique Rossi

**Affiliations:** 1 Universidade Federal da Bahia, Salvador, BA, Brasil.; 2 Hospital Geral Cleriston Andrade, Feira de Santana, BA, Brasil.; 3 Escola Bahiana de Medicina e Saúde Pública, Salvador, BA, Brasil.; 4 Campo Limpo Hospital, Campo Limpo, SP, Brasil.; 5 Hospital Instituto Dante Pazzanese de Cardiologia, São Paulo, SP, Brasil.

**Keywords:** vascular injury, pseudoaneurysm, hand injury, bleeding, lesões vasculares, pseudoaneurisma, traumatismo da mão, sangramento

## Abstract

Pseudoaneurysm of the palmar arch is a rare entity. Diagnosis is dependent on high clinical suspicion. We present a case referred to the emergency department, with a history of glass penetrating trauma to the palmar surface with a pulsatile mass and jet bleeding. Doppler ultrasound evidenced a partially thrombosed pseudoaneurysm. A CT angiography examination showed a saccular formation arising from the superficial palmar arch. A conventional surgical approach was indicated. A clinical suspicion must be ventured to arrive at the correct diagnosis. Imaging modalities are needed to identify the pseudoaneurysm and plan the treatment course. Nonetheless, the sequence of diagnosis is individual, because further evaluation with different imaging methods may not change the rationale for the intervention. In our experience, conventional surgical removal is preferable, due to its safety and well-established outcomes.

## Resumo

O pseudoaneurisma do arco palmar é uma entidade rara, cujo diagnóstico depende de alta suspeição clínica. Apresentamos o caso de um paciente encaminhado ao pronto-socorro com história de traumatismo penetrante por vidro na face palmar, com massa pulsátil e sangramento em jato. A ultrassonografia com Doppler evidenciou pseudoaneurisma parcialmente trombosado, e a angiotomografia demonstrou formação sacular originada do arco palmar superficial. Uma abordagem cirúrgica convencional foi indicada. Para prosseguir com o diagnóstico correto, essa suspeita clínica deve ser aventada. Modalidades de imagem são necessárias para identificar o pseudoaneurisma e planejar o curso do tratamento. No entanto, a sequência diagnóstica é individual, pois uma avaliação mais aprofundada, com diferentes métodos de imagem, pode não alterar o racional da intervenção. Em nossa experiência, a remoção cirúrgica convencional é preferível, visto sua segurança e seus resultados bem estabelecidos.

## INTRODUCTION

Despite the high incidence of hand trauma, pseudoaneurysm of the palmar arch is a rare entity with only a few cases described in the literature.^1,2^ After penetrating injury to the hand, microscopic defects within the arterial architecture can lead to pseudoaneurysm formation, also known as false aneurysm.^1-5^ Diagnosis is dependent on high clinical suspicion of the disease, especially in the presence of a pulsatile mass and appropriate imaging modalities.^1,4^ We present the case of a patient with a pseudoaneurysm of the superficial palmar arch following penetrating trauma who was treated surgically.

### Part 1: clinical case

A 25-year-old man was referred to the emergency department, with a history of glass penetrating trauma to the palmar surface of the left hand 10 days earlier. At that time, plain radiographic images showed no evidence of foreign body, bone injury, or any other abnormality. The patient was initially treated with a primary suture in a primary hospital. He developed severe pain, limited movement of the 1st and 2nd ipsilateral fingers, and jet bleeding at the site of previous suturing for two days. On physical examination, a pulsatile hematoma was observed in the left palmar region at the site of the previous suture, approximately 20 mm in diameter, with radial and ulnar pulses present, normal sensation, and capillary refill in his fingers. Allen’s test was performed, showing deep palmar arch patency.

**Figure 1:**
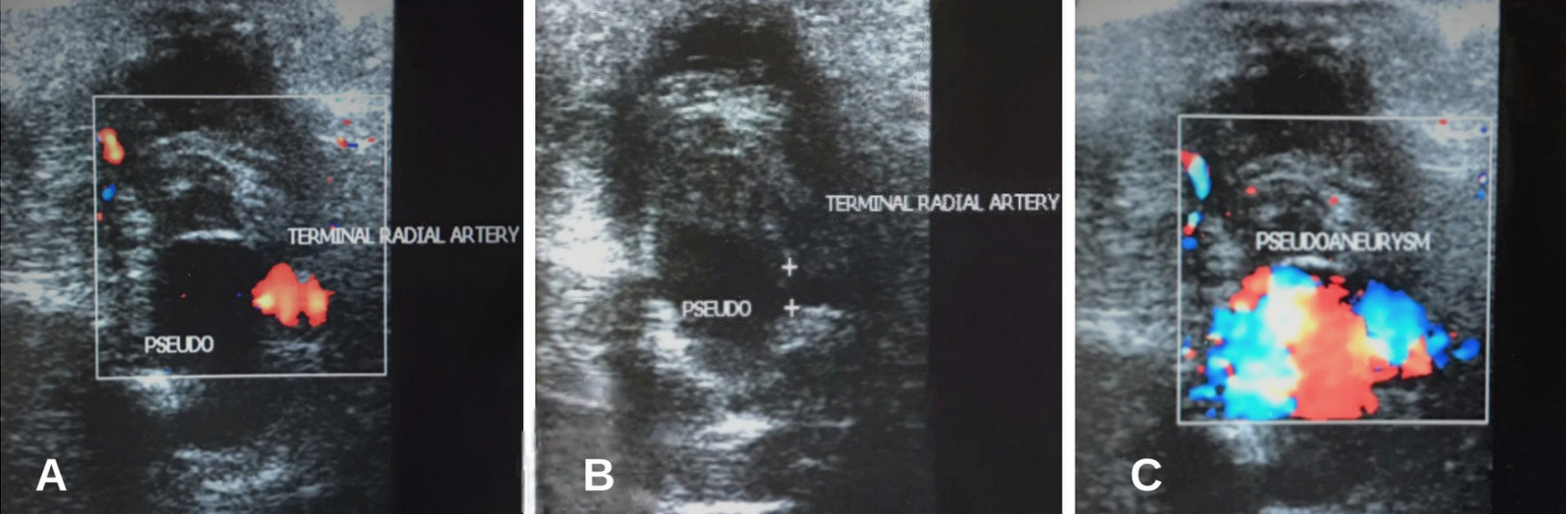
Ultrasound demonstrating superficial palmar arch pseudoaneurysm. (A) Preserved blood flow through the superficial palmar arch seen with Color-Doppler; (B) initial portion of the superficial palmar arch with a defect in the vessel wall allowing blood to pass to the adjacent soft tissues; (C) turbulent blood flow in the region of the superficial palmar arch pseudoaneurysm (Yin-Yang sign).

**Figure 2:**
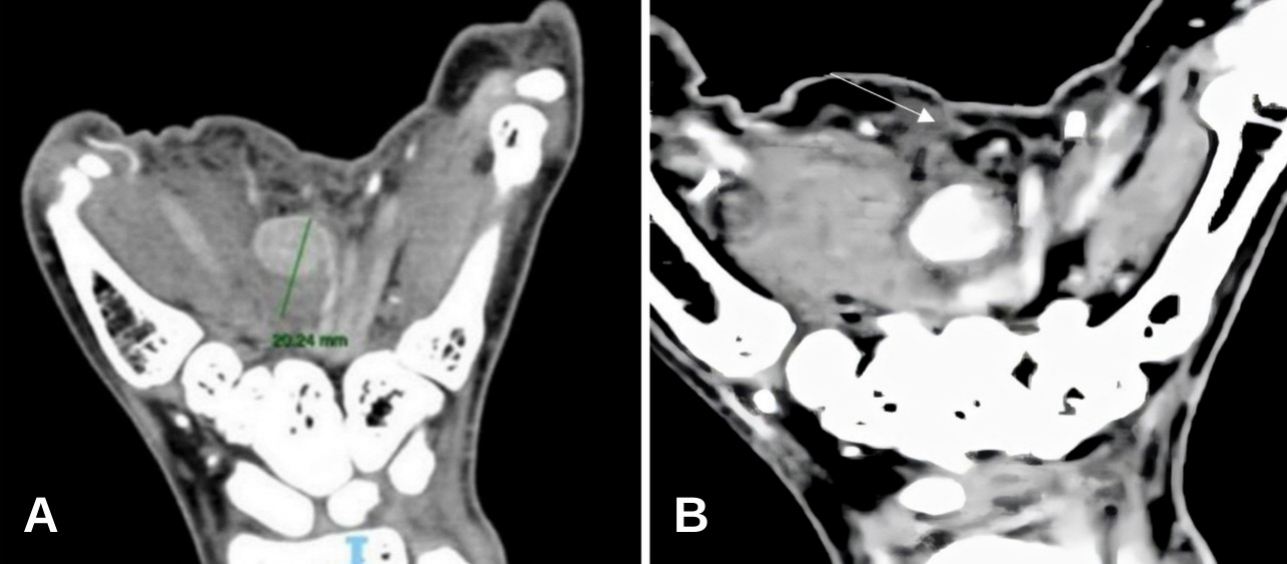
(A) Computed tomography angiogram showing a saccular formation in the superficial palmar arch, at the level of the second and third metacarpals, measuring up to 20 mm; (B) contrast flow in the pseudoaneurysm. Note the short distance from the palmar surface (white arrow).

A Doppler ultrasound of the left hand was requested (Figure 1A). A pseudoaneurysm was seen with a 20 mm diameter and a 6 mm short and broad neck, partially thrombosed (Figure 1B and 1C). The patient underwent computed tomographic (CT) angiography of the left hand, showing a saccular formation arising from the superficial palmar arch, at the level of the second and third metacarpals, measuring up to 20 mm in post-contrast enhancement images (Figure 2A and 2B).

### Part 2: what was done?

**Figure 3:**
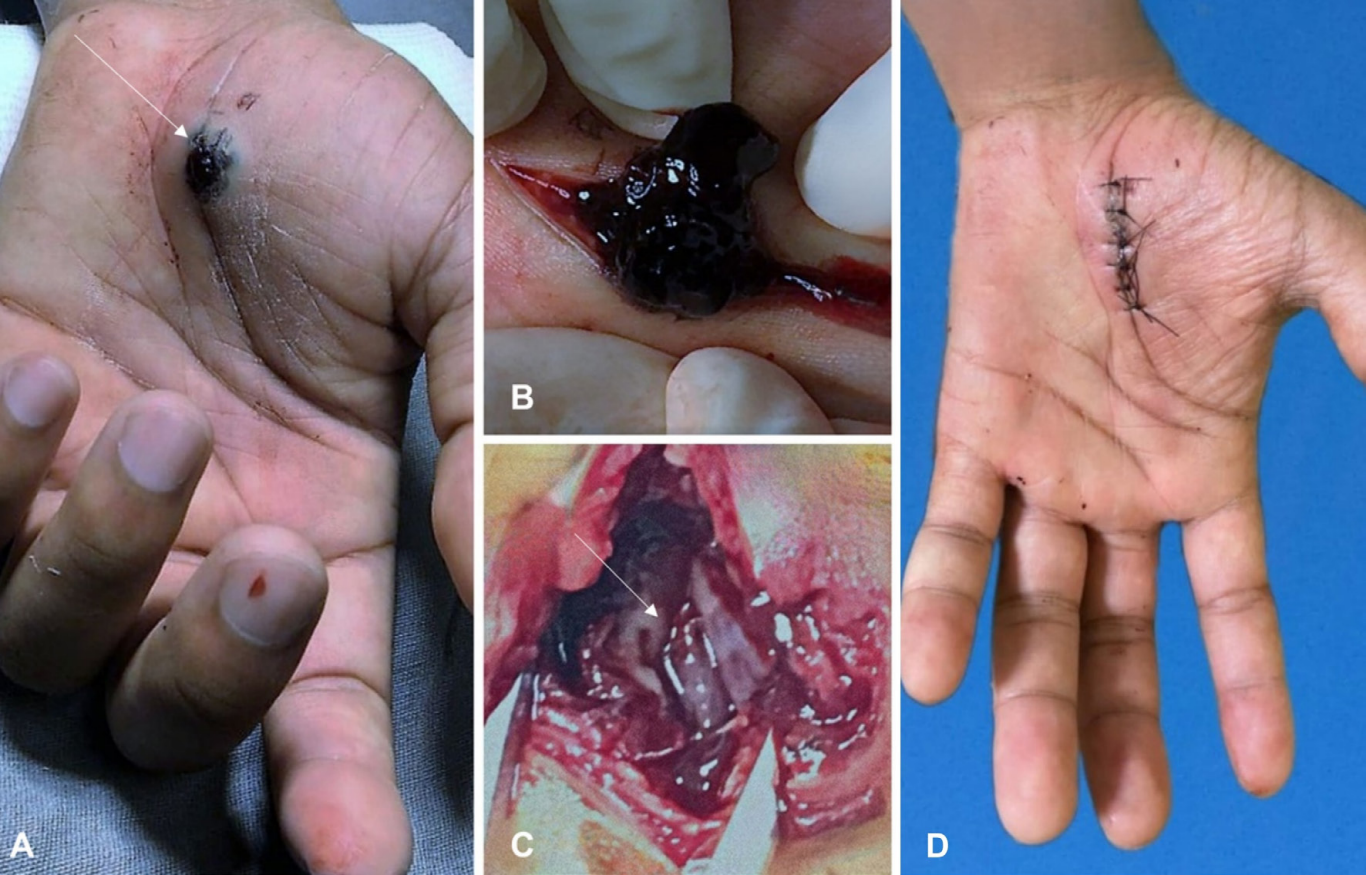
(A) Pulsatile hematoma in the palmar surface of the left hand. Note the jet bleed point (white arrow); (B) exposure of superficial palmar arch pseudoaneurysm thrombus; (C) complete removal of the pseudoaneurysm with suture of the injured artery. Note the superficial palmar artery (white arrow). (D) 15 days postoperatively. Wound with good evolution and without pulsatile mass, and with perfused hand.

A conventional surgical approach was indicated. The patient underwent local anesthesia and sedation, followed by a semicircular incision in the palm of the left hand and dissection of the pseudoaneurysm, with thrombus removal (Figure 3A and 3B). The point of injury was identified in the superficial palmar arterial arch, compromising less than 50% of the artery’s circumference. Primary suture of the injured artery was performed and distal perfusion was ensured (Figure 3C). No anticoagulation was administrated during or after surgery. The patient had an uneventful postoperative course with no signs of ischemia and was discharged home on the second day (Figure 3D). After 6 months’ follow-up based on clinical assessment exclusively, the patient presented with regular wound healing and was symptom-free (normal hand sensitivity and mobility and free from paresthesia).

Written informed consent was obtained from the patient and the study was approved by the Hospital Medical Care ethics committee, with reference number 4.451.766.

## DISCUSSION

**Figure 4:**
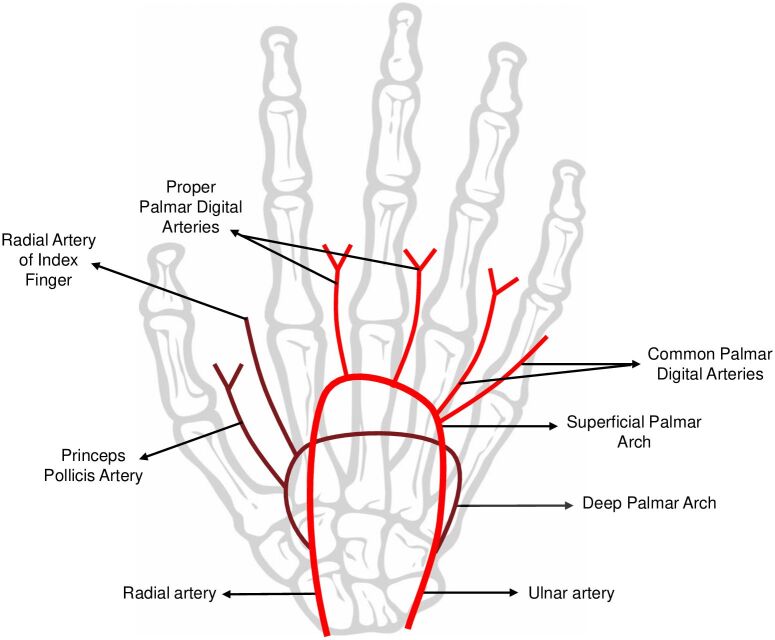
Representative image from superficial and deep palmar arch anatomy. The arrow shows the closest site to the vascular injury from the reported case.

The blood supply to the hand is predominantly formed of anastomosing vascular networks known as the superficial palmar arch (SPA) and the deep palmar arch (DPA).^6^ The SPA is commonly formed by collateral circulation between the ulnar artery (UA) and the superficial branch from the radial artery (RA), while the UA is the main contributor to the SPA^7^ (Figure 4). The SPA exhibits a high prevalence of anatomical variation, which stems from embryologic development,^6^ leading to a highly variable pattern of mechanical injuries along its course through the palmar region. The SPA curves laterally superficial to the long flexor tendons and deep to the palmar aponeurosis.^7^

Pseudoaneurysms of the superficial palmar arch are rare. There are only a few cases described in the literature.^3,8^ This entity is most frequently reported after penetrating trauma to the artery due to disruption of the arterial wall resulting in bleeding and formation of an adjacent hematoma, which is later surrounded by fibrous tissue, creating a continuous pocket with the artery. Compared with the native vessel, the pseudoaneurysm is more susceptible to rupture, although rupture is uncommon.^2^ Regardless of type, pseudoaneurysms are very rare in adults, and extremely rare in the pediatric population.^9^ They mostly occur as a result of penetrating hand trauma, repeated blunt trauma, infections, connective tissue diseases, vasculitis, and even congenital defects.^8,9^

Clinical presentation can include local pain, swelling, and a pulsatile mass in the palm near the site of injury, although hemorrhage is a rare presentation.^3,10^ Complications can include infection, arterial occlusion, nerve compression, abscess formation, and rarely, bone erosion.^2,5,10,11^ In this case, jet bleeding was present 10 days after trauma, which is an uncommon presentation.

Due to its rarity, differential diagnosis can be challenging. Lipomas, incision cysts, and fibromas must be excluded to arrive at the correct diagnosis.^1^ In a post-traumatic scenario, foreign body retention and abscess formation should be taken into consideration, but pulsation of the mass should raise suspicion of the presence of a false aneurysm, especially if it arises progressively.^4^

Imaging approaches to these lesions can be a matter of debate and there is a lack of studies with large samples. Ultrasonography is typically used as the initial diagnostic imaging modality and angiography is used to evaluate collateral blood flow.^3,5^ CT angiography can also be used, although magnetic resonance angiography is useful and does not need any contrast medium. These imaging methods are effective for assessment of the vascular anatomy, characteristics, and location of the pseudoaneurysm. However, the diagnostic sequence is individual, because further evaluation with different imaging methods may not change the rationale for intervention. Bouvet at al. proposed an imaging algorithm for suspected hand aneurysm.^8^ After an aneurysm is suspected, the first method should be Doppler ultrasound, which provides quick access, with reproducible technique, for positive diagnosis of aneurysm or pseudoaneurysm and for differential diagnosis.^8^ If the suspicion is confirmed, the second step is to assess whether there is acute ischemia or not. In cases presenting with acute ischemia, arteriogram is the next diagnostic step, remaining the gold standard for confirmation of arterial pseudoaneurysm, especially when there are other vascular abnormalities. It is considered a fundamental step prior to surgery and an exhaustive arterial angiogram is recommended, exploring the proximal arteries in order to exclude other sites of atheromatous embolism.^12^ If there is no evidence of acute ischemia, CT/MR angiography should be performed.

In this case, the patient underwent CT angiography because there was no sign of acute ischemia and patency of the deep palmar arch had been confirmed. Questions were raised about the sequence of imaging methods needed to evaluate the lesion, since further evaluation with different imaging modalities may not change the rationale for intervention and may not provide additional important anatomical details that CT angiography had not shown.

Surgical excision and vascular reconstruction are most often performed to ensure distal perfusion and avoid complications.^5,10,11^ Arteries have been repaired or resected, with or without bypass surgery.^2^ Reconstruction with end-to-end anastomosis using microsurgery has also been described.^1^ Restoration of the blood flow is preferable, especially in children.^1^ Uncomplicated, small, stable pseudoaneurysms can be managed conservatively with compression bandages and close follow-up.^13^ However, this could be dangerous due to the risk of distal embolization with subsequent occlusion of the run-off arteries. Ultrasound-guided thrombin injection has been found to be an effective, time-saving, and safe procedure for treatment of pseudoaneurysms,^2,8,14,15^ but it should be avoided as treatment for cases with arteriovenous fistula or when the pseudoaneurysm neck cannot be seen. Our patient was successfully treated with a conventional surgical approach with primary suture, the deep palmar arch was anatomically intact, and the damage to the superficial palmar arch was less than 50% of the vessel, with evidence of good perfusion.

Despite its rarity, pseudoaneurysm of the superficial palmar arch is a serious condition and can lead to severe symptoms and complications in patients after a palmar trauma. For correct diagnosis, suspicion of this condition should be investigated in steps to exclude differential diagnoses, proceeding with the correct imaging modalities. This is important, since there is no strong evidence in the literature on the diagnostic imaging sequence and the decision depends on the singularities of each case. We prefer conventional surgical treatment, due to its safety and well-recognized outcomes. However, other non-invasive treatments have been described in the literature with safety.
